# *In vitro* activity of gepotidacin and comparator antimicrobials against isolates of nontuberculous mycobacteria (NTM)

**DOI:** 10.1128/aac.01684-23

**Published:** 2024-04-24

**Authors:** Barbara A. Brown-Elliott, Georgie Bush, M. Dolores Hughes, Eliana Rodriguez, Chase A. Weikel, Sharon B. Min, Richard J. Wallace

**Affiliations:** 1 The University of Texas Health Science Center at Tyler, Mycobacteria/Nocardia Laboratory, The University of Texas at Tyler School of Medicine, Tyler, Texas, USA; 2 Department of Infectious Diseases, GSK, Collegeville, Pennsylvania, USA; Bill & Melinda Gates Medical Research Institute, Cambridge, Massachusetts, USA

**Keywords:** gepotidacin, nontuberculous mycobacteria, rapidly growing mycobacteria, slowly growing mycobacteria, antimicrobial activity

## Abstract

Novel antimicrobials are needed to treat rising nontuberculous mycobacteria (NTM) infections. Using standard broth microdilution methods, 68 NTM isolates were tested against gepotidacin, a new, first-in-class, oral triazaacenaphthylene bacterial topoisomerase inhibitor. MICs varied (0.25 to >64 µg/mL) with the lowest being *M. fortuitum* complex (0.25–8 µg/mL), *M. mucogenicum* complex (1–2 µg/mL), *M. kansasii* (0.25–8 µg/mL), and *M. marinum* (4–16 µg/mL). Testing greater numbers of some species is suggested to better understand gepotidacin activity against NTM.

## INTRODUCTION

Nontuberculous mycobacterial (NTM) infections are increasing globally. *Mycobacterium avium* complex (MAC) and the *Mycobacterium abscessus* subspecies are two of the most antimicrobial-resistant taxa (1–6). Therapeutic antimicrobials are limited due to antibiotic resistance mechanisms such as the erythromycin resistance methylase (*erm*) gene and by intolerability of the patient to the treatment regimen (1–11).

Most NTM treatments involve lengthy multi-antimicrobial regimens including potentially toxic agents such as cefoxitin, imipenem, tigecycline, and amikacin which may cause serious adverse events (5–10, 12). Fluoroquinolones (ciprofloxacin and moxifloxacin) may be effective against some clinically relevant NTM such as *M. fortuitum* complex*, M. mucogenicum* complex, and *M. kansasii* but are ineffective against others like *M. abscessus* species which are resistant to most oral antimicrobials (1–4, 12). Because rapid development of resistance can occur, quinolone monotherapies are not recommended (13).

Gepotidacin is a first-in-class, oral triazaacenaphthylene antimicrobial that selectively inhibits bacterial DNA gyrase and topoisomerase IV by a unique mechanism of action not utilized by any currently approved therapeutic agent (14). Gepotidacin has *in vitro* activity against target bacterial pathogens including most fluoroquinolone-resistant strains and has recently completed phase 3 clinical studies for uncomplicated urinary tract infections (uUTI) and urogenital uncomplicated gonorrhea (uGC) (15–23). we aimed to investigate the *in vitro* activity of gepotidacin against various NTM species. This work was approved by the Institutional Review Board at the University of Texas Health Science Center at Tyler.

Isolates tested were clinical NTM submitted to the Mycobacterial/Nocardia Laboratory in the past 5 years for antimicrobial susceptibility testing (AST) and species identification using gene sequencing, as previously described (4, 24–28).

All MIC testing was performed in duplicate by broth microdilution (BMD) in accordance with CLSI guidelines (27, 28). All duplicate MICs were either equal or within one twofold dilution of each other (and within the same interpretive criteria i.e., S, susceptible; I, intermediate; R, resistant). Quality control (QC) was performed according to CLSI guidance (27–29). Reference strains were obtained from the American Type culture Collection (ATCC) (27, 28). There are no CLSI-approved QC ranges for gepotidacin against NTM (27, 28). Only amikacin, clarithromycin, linezolid, and moxifloxacin MICs were reported for MAC isolates as per CLSI/ATS/IDSA recommendations (5, 6, 27, 28).

AST of RGM was performed in CAMHB using custom-prepared available 96-well microtiter panels (Thermo Fisher Scientific, Cleveland, OH) and incubated at 30°C for 3–5 days until adequate growth was visible macroscopically in the control well. Slowly growing mycobacteria (SGM) were tested in CAMHB +oleic acid-albumin-dextrose-catalase (OADC) incubated at 35°C for 7–14 days until adequate growth was present in the control well (27, 28).

For rapidly growing mycobacteria (RGM) isolates (*n* = 39), the gepotidacin MIC range was 0.25 to >64 µg/mL (Table 1). The lowest gepotidacin MICs were against *M. fortuitum* complex (*n* = 13) [*M. fortuitum* (*n* = 7), *M. porcinum*/*boenickei* (*n* = 2), and *M. senegalense* (*n* = 4)]. The two isolates of *M. porcinum/boenickei* had gepotidacin MICs of 0.25 and 0.5 µg/mL. *M. fortuitum* and *M. senegalense* had gepotidacin MICs of 1–2 µg/mL and 1–8 µg/mL, respectively (Table 1). One *M. fortuitum* isolate had a ciprofloxacin MIC of 1 µg/mL. Although within the susceptible range for ciprofloxacin, in our observations (unpublished data), MICs > 0.12 µg/mL for this species are suggestive of previous treatment with a fluoroquinolone. Moreover, the gepotidacin MIC for this isolate was 2 µg/mL. Gepotidacin MICs of 2 µg/mL were also observed in two other isolates of *M. fortuitum* which had ciprofloxacin MICs ≤ 0.12 µg/ml. *M. mucogenicum* complex (*n* = 3) and *M. goodii* (*n* = 2) had gepotidacin MICs ranging from 1 to 2 µg/mL and corresponding ciprofloxacin and moxifloxacin MICs ranging from ≤0.12 to 1 µg/mL.

**TABLE 1:** MIC values (µg/mL) of gepotidacin and comparator antimicrobials against 39 isolates of rapidly growing mycobacteria^*a*^

Species/complex subspecies	No. of isolates	MIC (µg/mL)	GEP	CIP	MXF	DOX	MIN	TGC	AMK	FOX	SXT	LZD	IPM	CLR	TOB^*b*^
*M. abscessus* subsp. *abscessus*	13	MIC range	32 to >64	2 to >4	4 to >8	16 to >16	8 to >8	0.03–0.25	4–16	16–64	1/19 to >4/76	4–32	4–16	2 to >16	NA
		MIC_50_	32	>4	8	>16	>8	0.06	8	32	4/76 to >4/76	16	8	>16	NA
		MIC_90_	>64	>4	>8	>16	>8	0.12	16	64	4/76 to >4/76	32	16	>16	NA
*M. abscessus* subsp. *massiliense*	2	MIC range	32–64	>4	4 to >8	>16	4 to >8	0.06–0.12	8–16	32	4/76	8	8–16	0.5–2	NA
*M. chelonae*	4	MIC range	8–32	2–4	2–4	>16	>8	0.03–0.12	4–32	>128	2/38 to >4/76	4–8	16– 32	1– 4	≤1–2
*M. immunogenum*	2	MIC range	16–32	2	4	16 to >16	>8	0.12	8	>128	4/76 to >4/76	8	16	2	16 to >16
*M. fortuitum^d^*	7	MIC range	1–2	≤0.12–1	≤0.25	≤0.12 to >16	≤1–16	≤0.015–0.06	≤1–2	32	≤0.25/4.75 to 2/38	≤1– 16	≤2–4	8 to >16	NA
*M. porcinum/boenickei* complex	2	MIC range	0.25–0.5	≤0.12–0.5	≤0.25	16	4–8	≤0.015	≤1	16	≤0.25/4.75 to 1/19	≤1–2	≤2–8	16	NA
*M. senegalense^c^*	4	MIC range	1–8	≤0.5 to >4	≤0.25–0.5	≤0.12	≤1	≤0.015–0.06	≤1	16–32	≤0.5/9.5 to 2/38	≤1–4	≤2–4	0.5–1	NA
*M. mucogenicum* complex^*e*^	3	MIC range	1–2	≤0.12–1	≤0.25–0.5	≤0.12 to >16	≤1 to >8	0.06–0.12	≤1–2	≤8–16	≤0.25/4.75	≤1	≤2	1–2	NA
*M. goodii*	2	MIC range	1–2	≤0.12–0.25	≤0.25	≤0.12	≤1	≤0.015–0.03	≤1	64 to >128	≤0.25/4.75	≤1	≤2–4	16 to >16	NA

^*a*^ Abbreviations: NA= not available; GEP = gepotidacin; CIP = ciprofloxacin; MXF = moxifloxacin; DOX = doxycycline; MIN = minocycline; TGC = tigecycline; AMK = amikacin; FOX = cefoxitin; SXT = trimethoprim sulfamethoxazole; LZD = linezolid; IPM = imipenem; CLR = clarithromycin; TOB = tobramycin.

^*b*^ Tobramycin MIC not reported per CLSI except for *M. chelonae* (≤2 µg/mL) and *M. immunogenum* (≥8 µg/mL).

^*c*^ Includes 1 CIP-resistant (>4µg/mL) isolate.

^*d*^ *M. fortuitum* complex includes *M. fortuitum*, *M. porcinum*/*boenickei* complex and *M. senegalense* plus other species not tested.

^*e*^ *M. mucogenicum* complex includes *M. mucogenicum* and *M. phocaicum*.

The highest gepotidacin MICs among RGM were noted with the *M. abscessus* subsp. *abscessus* (*n* = 13, MIC_50_ = 32 µg/mL; MIC_90_ = >64 µg/mL). Similarly, the two isolates of subsp. *massiliense* had gepotidacin MICs of 32 and 64 µg/mL. MICs for four isolates of *M. chelonae* (8–32 µg/mL) and two isolates of *M. immunogenum* (16 and 32 µg/mL) were only slightly lower than *M. abscessus subsp. abscessus* (32 to > 64 µg/mL).

For SGM isolates (*n* = 29), the gepotidacin MIC range was 0.25–>64 µg/mL and was lowest against *M*. *kansasii* (*n* = 4, MIC range 0.25–8 µg/mL) and *M. marinum* (*n* = 5, MIC range 4–16 µg/mL) (Table 2). Ciprofloxacin and moxifloxacin MICs were 0.25–1 µg/mL and ≤0.12 µg/mL, respectively, for *M. kansasii*. A range of 1–4 µg/mL for ciprofloxacin and 0.5–2 µg/mL for moxifloxacin was observed for *M. marinum* isolates.

**TABLE 2:** MIC values (µg/mL) of gepotidacin and comparator antimicrobials against 29 isolates of slowly growing mycobacteria including *Mycobacterium avium* complex^*a*^

Species	No. of isolates	MIC result type (µg/mL)	GEP	CIP	MXF	DOX	AMK	SXT	LZD	CLR	RFB	RIF
*M. kansasii*	4	MIC range	0.25–8	0.25–1	≤0.12	0.5–2	≤1–2	≤0.12/2.38 to 0.5/9.5	≤1	≤0.06 to >64	≤0.25	≤0.12
*M. marinum*	5	MIC range	4–16	1–4	0.5–2	0.5–2	≤1	0.25/4.75 to 0.5/9.5	≤1–2	0.5–1	≤0.25	0.25–0.5
*M. simiae*	5	MIC range	64 to >64	4 to >16	2 to >8	16 to >16	8–16	0.5/9.5 to 4/76	8–32	1–16	4 to >8	8 to >8
*M. arupense*	4	MIC range	>64	16 to >16	>8	8 to >16	16–64	≤0.12/2.38 to 1/19	≤1–32	0.12–4	≤0.25–1	2 to >8
*M. avium* complex	11	MIC range	4 to >64	0.25 to >16	≤0.12–8	NA	4–32	NA	≤1–32	0.25–4	NA	NA
		MIC_50_	>64	>16	4	NA	16	NA	32	2	NA	NA
		MIC_90_	>64	>16	8	NA	32	NA	32	4	NA	NA

^*a*^ Abbreviations: NA= not applicable; GEP = gepotidacin; CIP = ciprofloxacin; MXF = moxifloxacin; DOX = doxycycline; AMK = amikacin, SXT = trimethoprim/sulfamethoxazole; LZD = linezolid; CLR = clarithromycin; RFB = rifabutin; RIF = rifampin.

The highest gepotidacin MICs were seen against *M. arupense* (*n* = 4) and *M. simiae* (*n* = 5) and ranged from 64 to >64 µg/mL. In addition, all but one of these were ciprofloxacin resistant and moxifloxacin resistant. One isolate of *M. simiae* was the moxifloxacin intermediate.

Eleven MAC isolates were analyzed. The gepotidacin MIC range against MAC was 4 to >64 µg/mL. Two isolates had lower gepotidacin MICs (4 and 16 µg/mL) with corresponding ciprofloxacin and moxifloxacin MICs of 0.25–1 µg/mL and ≤0.12–0.5 µg/mL, respectively. Overall, ciprofloxacin and moxifloxacin MICs ranged from 0.25 to >16 µg/mL and ≤0.12–8 µg/mL, respectively. All comparator antimicrobial MICs were within CLSI-accepted QC ranges for SGM and RGM (27, 28). In addition, all gepotidacin QC results were within CLSI-accepted MIC QC ranges for *Staphylococcus aureus* ATCC 29213 and *Enterococcus faecalis* ATCC 29212 (29).

To our knowledge, only this study and another by Ahmad et al. report on *in vitro* NTM susceptibility to gepotidacin (30). Our current study showed gepotidacin MICs against clinical NTM isolates to be variable and species specific with limited or no activity against the two major groups responsible for human infections (*M. abscessus* subspecies and MAC). In addition, gepotidacin MICs were higher or equivalent to those of ciprofloxacin and moxifloxacin and although gepotidacin has demonstrated efficacy against other bacterial strains, our current study does not show *in vitro* activity against most clinically significant NTM (15, 19, 21, 22). Unfortunately, the results from Ahmad et al. cannot be reliably compared with this study due to the methodological differences between the studies (i.e., AST methods not consistent with current CLSI guidelines) (27, 28, 30). To better understand the full extent of gepotidacin activity against NTM, additional studies with larger isolate collections would be required.
